# One-Pot Synthesis of Silica-Coated Gold Nanostructures Loaded with Cyanine 5.5 for Cell Imaging by SERS Spectroscopy †

**DOI:** 10.3390/nano13071267

**Published:** 2023-04-03

**Authors:** Aleksei N. Smirnov, Simar F. Aslanov, Denis V. Danilov, Olga Yu. Kurapova, Elena V. Solovyeva

**Affiliations:** 1 Chemistry Institute, Saint-Petersburg State University, 26 Universitetsky Pr., Peterhof, 198504 Saint-Petersburg, Russia; alexnicksmirnow@gmail.com (A.N.S.); aslanov.simar@gmail.com (S.F.A.); o.y.kurapova@spbu.ru (O.Y.K.); 2 Interdisciplinary Center for Nanotechnology, Saint-Petersburg State University, 1 Ulianovskaya Str., Peterhof, 198504 Saint-Petersburg, Russia; d.danilov@spbu.ru

**Keywords:** gold nanoparticles, silicon oxide, one-pot synthesis, tag, SERS, bioimaging, cyanine

## Abstract

Anisotropic gold nanoparticles have been recognized as promising agents for medical diagnostics and cancer therapy due to their wide functionality, photothermal effect, and ability for optical signal amplification in the near-infrared range. In this work, a simple and rapid method for the preparation of bone-shaped gold nanoparticles coated with a dye-impregnated silica shell with an aminated surface is proposed. The possibility of further functionalization the nanostructures with a delivery vector using folic acid as an example is demonstrated. The average size of the resulting tags does not exceed 70 nm, meeting the criteria of cell endocytosis. The prepared tags exhibit surface-enhanced Raman scattering (SERS) spectra at excitation with lasers of 632.8 and 785 nm. Cell imaging is performed on HeLa cells based on the most pronounced SERS bands as a tracking signal. The obtained images, along with scanning electron microscopy of cell samples, revealed the tendency of tags to agglomerate during endocytosis followed by the “hot spots” effect. To evaluate the toxic and proliferative effect of the nanotags, an MTT assay was performed with two HeLa and HEP G2 cell lines. The results revealed higher viability for HEP G2 cells.

## 1. Introduction

Methods of bioanalytics and medical diagnostics based on molecular spectroscopy have become an integral part of modern life. The interaction of light with matter is used in numerous chemical analyzes based on the “antigen–antibody” specificity or in the diagnostics of internal organs using physical methods. However, the improvement of sensitivity and reduction of detection limit of optical methods in medical diagnostics are limited by the interference of the biological matrix. Increasing the recorded signal intensity is assumed to be one of the most efficient ways to overcome this challenge.

Since the discovery of the plasmon resonance effect, noble metal nanoparticles have been considered as substrates that provide the optical signal enhancement from a variety of organic compounds. The examples of their in vitro, ex vivo, and in vivo applications are already known [1,2,3]. Plasmonic nanoparticles give a strong amplification of Raman scattering known as surface-enhanced Raman scattering (SERS) and have a less pronounced and more complex effect on fluorescence. Despite plasmonic nanoparticles are more sophisticated and multipurpose agents, their use in fluorescence spectroscopy remains questionable due to the quenching effect competing with the electromagnetic enhancement. The relationship between these effects strongly depends on the distance between the fluorophore and the plasmonic core [4,5,6]. Usually, fluorescence is quenched when the distance between the fluorophore and metal core is less than 2–5 nm and complete disappearance of the emission can be observed directly on the surface. At distances greater than 5–10 nm, an electromagnetic enhancement of fluorescence can occur. The ability of plasmonic nanoparticles to enhance either Raman scattering or fluorescence is deeply connected to their absorption cross-section, which is orders of magnitude greater than that of small molecules [7,8]. This feature is explored not only in analytical chemistry and medicine but also in material science for alternative energy sources where noble metal nanoparticles play the role of sensitizer in the solar cells [9].

The advantage of plasmonic nanoparticles is not limited to imaging performance, and they can also be used for therapeutic purposes due to the photothermal effect. Local heating of nanoparticles located in the malignant tumors with a laser can destroy the cancer cells [10]. However, there are two major problems of noble metal nanoparticles application in photothermal therapy that should be addressed: (i) the difficulty of racking the nanoparticles in the depth of tissue and (ii) the insufficient selectivity of nanoparticles localization. To overcome these problems, complex nanotags have been actively developed consisting of a metal nanoparticle, a shell functionalized with molecular labels and a delivery vector.

Coating plasmonic nanoparticles with a shell also helps to expand greatly their application in the field of diagnostics and therapy. A wide variety of hybrid gold core-shell systems exhibiting more rich physical and chemical properties is known [11,12]. Namely, a combination of gold nanoparticles with materials exhibiting paramagnetic properties is promising for magneto-optical imaging [13,14,15,16]. Loading the shell with anticancer chemicals or specific oligonucleotide inhibitors is favorable for targeted chemotherapy of cancer with reduced side effects [17,18,19]. Gold nanoparticles with a shell, including specific antigens or immune-stimulating agents, are advantageous for immunotherapy of cancer [20].

In addition to expanded functionality, coating gold nanoparticles with a shell improves their biocompatibility and stability, preventing the degradation in various media. Silica is one of the most popular and convenient materials for use as a protective shell for different inorganic nanoparticles [21,22,23,24]. Moreover, the silica surface can be additionally functionalized, opening new frontiers for covalent immobilization of various molecules, in particular, delivery vectors [25,26]. Another great advantage of using silica is that it has no impact on the dye fluorescence response in comparison with other coatings such as bovine serum albumin or poly(allylamine hydrochloride). Thus, silica is one of the most promising surface modifiers for plasmonic nanotags, whose operating principle is based on SERS or fluorescence. It is important to note that the thickness of silica shell can be easily tuned by varying tetraethoxysilane (TEOS) concentration in the reaction [27,28].

The Stober method is the most popular approach to coat the metal nanoparticles with a silica shell. This method was initially developed to produce silica particles without any nuclei [29,30]. In the Stober method, tetraethoxysilane undergoes hydrolysis, and its incomplete course leads to the formation of a mesoporous structure. Such a structure can be consciously used to localize various molecules, such as doxorubicin, which is a model anticancer chemical [31]. Another method of silica coating is called “active silicon oxide” (active silica), in which sodium silicate is subjected to proton exchange for activation followed by silica formation under hydrolysis conditions [32]. Although this approach is more laborious than the Stober method, it allows one to obtain thinner and denser shells (less than 2 nm). In all methods of coating with silica, successful binding of silane monomers to nanoparticle surfaces is required. One of the approaches of surface preparation for silane monomers immobilization involves the formation of a suitable zeta potential, which should be equal approximately −20 mV, according to the empirical data [33]. This value can be achieved by coating the nanoparticles with polyvinylpyrrolidone. In particular, Liz-Marzan et al. proposed a method for sequential recharge of CTAB-stabilized gold nanoparticles by stepwise coating with different polyelectrolytes with the last layer of polyvinylpyrrolidone [33].

Another approach for coating metal nanoparticles with a silica shell consists of the covalent adsorption of a silane derivative, such as mercaptopropyltrimethoxysilane (MPTMS), followed by hydrolysis which is performed in a manner similar with the Stober method. This approach has been criticized in the literature for the possibility of nanoparticle coagulation during the direct coating. Despite this, the simplicity and rapidity of coating metal nanoparticles with silica using MPTMS retains a high potential for this method, making it relevant and requiring revision.

In this work, an improved procedure is found for direct coating of gold nanoparticles with a dye-impregnated silica shell using MPTMS. The proposed technique is optimized in the reaction conditions and speed. An amount of MPTMS is adjusted to avoid the nanoparticle agglomeration during the first stage of surface functionalization. Second stages of silica shell growth and fluorophore impregnation are joined and performed in a one-pot regime with further pre-functionalization of shell surface for delivery vector immobilization. All together it has opened a simple and fast route for preparation of complex plasmonic nanotags suitable for cell imaging with SERS spectroscopy.

## 2. Materials and Methods

### 2.1. Chemicals

Cetyltrimethylammonium bromide (CTAB) (Sigma-Aldrich (Schnelldorf, Germany), 95%), hydrogen tetrachloroaurate (HAuCl_4_) (Alfa Aesar (Haverhill, MA, USA), 99%), sodium borohydride (NaBH_4_) (Sigma-Aldrich (Schnelldorf, Germany), 99%), ascorbic acid (Sigma-Aldrich (Schnelldorf, Germany), 99%), silver nitrate (AgNO_3_) (Sigma-Aldrich (Schnelldorf, Germany), 99%), cyanine 5.5 amine (Lumiprobe ((Moscow, Russia), 95%), triethoxysilane (TEOS) (Sigma-Aldrich (Schnelldorf, Germany), 99%), (3-mercaptopropyl)triethoxysilane (MPTMS) (Sigma-Aldrich (Schnelldorf, Germany), 99%), (3-aminopropyl)triethoxysilane (APTES) (Sigma-Aldrich (Schnelldorf, Germany), 99%), folic acid (FA) (Sigma-Aldrich (Schnelldorf, Germany), 99%), isopropyl alcohol (IPA) (Vekton (Saint-Petersburg, Russia), 98%), Dulbecco’s modified eagle’s medium (DMEM) (cat. no. ATCC 30-2002), fetal bovine serum (FBS) (Capricorn (Ebsdorfergrund, Germany)), trypsin-EDTA 0.25% (Capricorn TRY-3B (Ebsdorfergrund, Germany)), Dulbecco’s phosphate-buffered saline (Corning 21-031-CV (Glendale, Arizona, USA)), and antibiotic/antimycotic solution (100×, Capricorn AAS-B (Ebsdorfergrund, Germany)) were used without further purification. Glassware was washed with aqua regia and rinsed with deionized water before use.

### 2.2. Preparation of AuNB@Cy5.5@SiO_2_@FA Nanotags

**Figure 1:**
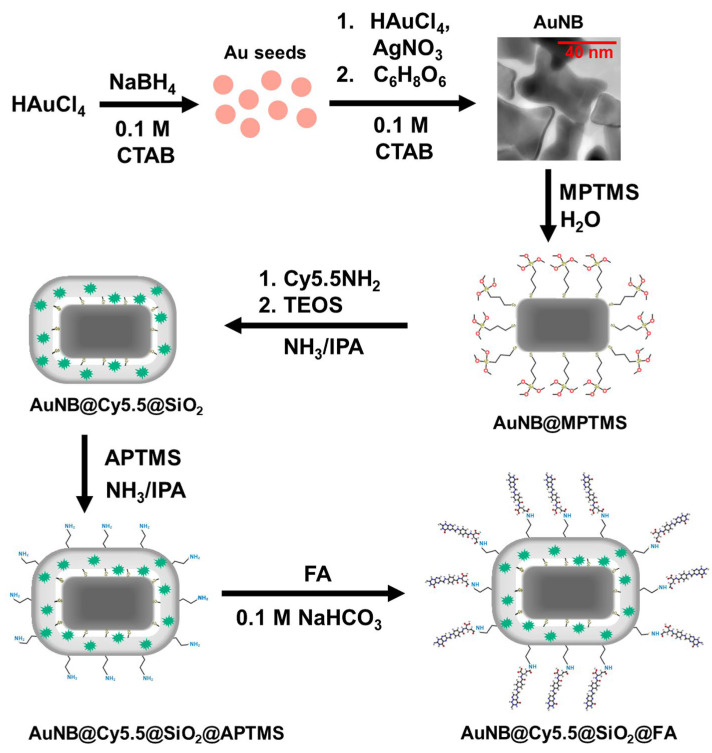
Scheme of the nanotags preparation including a synthesis of anisotropic gold nanoparticles, their labeling with a fluorophore, coating with a silica shell, and modification with a delivery vector.

Anisotropic gold nanoparticles (nanobones, AuNB) were obtained by a two-step seed-mediated method being a modification of the standard synthesis of gold nanorods [34,35]. In the method used, a concentration of ascorbic acid in the growth solution was increased, and the seeds were added immediately without keeping the reaction mixture for 2 h. Full procedure of the nanotags preparation is schematically presented in Figure 1 and includes all steps described below.

The seed solution was prepared by mixing 5 mL of 0.2 M CTAB solution with 5 mL of 0.1 mM HAuCl_4_. After that, 0.6 mL of 0.01 M ice-cold NaBH_4_ were added to the mixture. The color of solution changed from yellow to creamy brownish. The seed solution was used immediately after the observed color change (no later than 5 min after the preparation).

The growth solution was prepared by mixing 200 mL of 0.2 M CTAB solution with 10 mL of 10 mM AgNO_3_ under vigorous stirring followed by the addition of 200 mL of 1 mM HAuCl_4_. Then, 2.8 mL of 0.1 M ascorbic acid was added to the reaction mixture, followed by the addition of 0.552 mL of the seed solution. The reaction mixture was stirred for 48 h in a dark place at 25 °C. The obtained colloid had dark blue color. To remove excess CTAB, the resulting colloid was centrifuged step-wise at 10,000 and 8500 rcf, respectively.

To coat the gold nanoparticles with a silica shell, a solution of MPTMS in isopropanol with a concentration of 10^−4^ M was added to purified colloid in a ratio of 1 to 10. The mixture was stirred for 24 h. Then excess unreacted MPTMS was removed by centrifugation. Higher concentrations of MPTMS led to a decrease of nanoparticle stability. After MPTMS modification, the colloid was resuspended in a 20 times smaller volume of isopropanol than the original one. Next, the silane hydrolysis was carried out according to Stober method [30] with a TEOS concentration of 0.032 vol. % under stirring in a dark place overnight. Lower concentrations of TEOS resulted in the coagulation of nanoparticles. To incorporate the dye during a shell growth, a solution of cyanine 5.5 amine was added in the reaction vessel after TEOS with a concentration of 10^−4^ M.

Before the modification with a delivery vector, the surface of the gold nanoparticles was functionalized with amino groups by incubation overnight with 10^−4^ M solution of APTES in isopropanol. This solution was added a day after coating with silica in the one-pot mode. The latter was possible since the synthetic conditions for APTES modification are similar to the conditions for coating nanoparticles with silica. The silica-coated nanoparticles functionalized with APTES were transferred from isopropanol to water by double centrifugation with solvent decantation and resuspension in deionized water to 50 mL after the first centrifugation and to 10 mL after the second centrifugation. The synthesis of nanotags based on bone-shaped gold nanoparticles coated with a dye-impregnated silica shell with aminated surface was reproduced three times. The obtained nanotags showed stability up to six months at storage in a dark glass bottle at 4 °C.

Folic acid was chosen as a low molecular weight delivery vector to evaluate the antitumor applicability of the obtained nanotags. A solution of folic acid with a concentration of 10^−4^ M in 0.1 M NaHCO_3_ was added to the nanoparticles washed from isopropanol and excess APTES. The solvent with a basic pH was used to convert folic acid into the anionic form to optimize the conditions for its electrostatic interaction with the aminated surface. The excess non-immobilized folate species were removed by centrifugation.

### 2.3. UV-Vis Absorption Spectroscopy and Transmission Electron Microscopy and Energy Dispersive X-ray Analysis

The ultraviolet-visible (UV-Vis) absorption spectra of gold colloid were recorded on a UV-1800 spectrophotometer (Shimadzu (Kyoto, Japan)) with a spectral resolution of 1 nm. The spectra were measured at room temperature in a quartz cell with optical pathway of 1 cm.

Transmission electron microscopy (TEM) images of gold nanoparticles before and after shell coating were taken on a Libra 200FE electron microscope (Zeiss (Jena, Germany)) with an accelerating voltage of 200 kV. The colloid was applied dropwise (10 μL) onto the surface of carbon films, after which the samples were placed in a dark place for the solvent evaporation in air. TEM images were recorded from three random areas of each sample. Energy dispersive X-ray (EDX) spectra were measured with a X-Max Silicon Drift Detector (SDD) detector (Oxford Instruments Inc. (Abingdon, Oxfordshire, UK)). The line profiles for Si Kα, O Kα and Au Lα signals were recorded for nanotags coated by silica.

### 2.4. Scanning Electron Microscopy of Cell Samples

To prepare the samples for scanning electron microscopy (SEM) measurements, the cells were incubated overnight in 6-well plates containing glass coverslips at a seeding density of 4 × 10^5^ in standard DMEM cultural medium (DMEM with 10% FBS, glutamine, streptomycin, amphotericin B, penicillin). After that, the medium was removed and the wells were washed with phosphate-buffered saline and filled with 2 mL of DMEM containing 5 μg/mL of nanotags. After 2 h, the cells were washed three times with phosphate-buffered saline, the coverslips were transferred to a new 6-well plate and fixed with 4% formaldehyde in phosphate-buffered saline for 15 min. Stepwise dehydration was performed in 35/50/70/95/100% ethanol solutions with 5 min incubation after each step. Then coverslips were air-dried for 24 h, stained with carbon, and studied by SEM.

SEM images were obtained using an ORION microscope (Zeiss (Jena, Germany)) at an accelerating voltage of 10 kV in the scanning mode with a backscattered ion detector and an electron beam surface charge compensation system. The ion beam current was 262 pA.

### 2.5. Surface-Enhanced Raman Spectroscopy of Colloids and Cell Samples

SERS spectra of colloids were obtained at excitation with 532 and 785 nm lasers using a SENTERRA Raman spectrometer (Bruker (Billerica, MA, USA)), and with 632.8 nm laser using a LabRam HR-800 Raman spectrometer (Horiba Jobin Yvon (Palaiseau, France)). SERS spectra from the individual points of the cell monolayer and SERS maps of cell samples were obtained using a SENTERRA Raman spectrometer under excitation with 785 nm laser at a power of 25 mV and a signal accumulation time of 4 s in two repetitions.

To prepare the samples for SERS measurements, the cell lines were incubated overnight in a 6-well plate containing quartz coverslips at a seeding density of 4 × 10^5^ in DMEM. After that, the medium was removed and the wells were washed with phosphate-buffered saline and filled with 2 mL of DMEM containing 5 μg/mL of nanoparticles. After 2 h, the cells were washed three times with phosphate-buffered saline, the coverslips were transferred to a new 6-well plate and fixed with 4% formaldehyde in phosphate-buffered saline for 15 min, then washed again with buffer. Next, the coverslips were removed from the wells and sealed along the perimeter utilizing nail polish on glass slides, keeping the cell monolayer inside.

### 2.6. Cell Viability Assay

For the MTT assay with the nanotags, cervical epithelial carcinoma cell line HeLa was chosen as a folate-positive cell line and hepatocellular carcinoma cell line HEP G2 was chosen as an appropriate model system for the studies of liver metabolism. HeLa and HEP G2 cells were seeded in a 96-well plate at a density of 10,000 cells/well and incubated overnight with DMEM. After that, the medium was removed and the wells were filled with DMEM containing nanoparticle concentrations of 0.2, 1, and 5 μg/mL in five biological replicates, 16 wells were used for intact control cells. Cell viability was studied prior to and after functionalization of silica-coated nanoparticles with folic acid.

## 3. Results and Discussion

### 3.1. Morphology and Localized Surface Plasmon Resonance of AuNB@Cy5.5@SiO_2_@FA Nanotags

**Figure 2:**
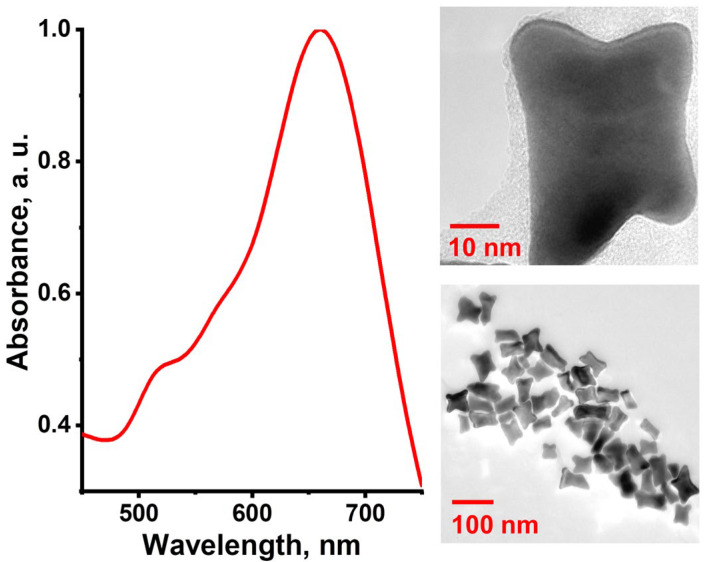
UV-Vis absorption spectrum and TEM images of bone-shaped gold nanoparticles.

The absorption spectrum and TEM images of the obtained gold nanoparticles are shown in Figure 2. As can be seen, the nanoparticles possess a bone-like shape, having “sags” on each of the short and long sides. Such a shape may be considered as a distorted nanorod crystalline structure. Higher concentration of ascorbic acid used in the growth solution leads to the more kinetically favorable occupation of crystalline facets by metal atoms. In comparison with the conventional synthesis of gold nanorods [36], the ascorbic acid–gold molar ratio is seven times higher in the case of nanobones. The use of seeds immediately after their preparation makes it possible to avoid the presence of “overgrown” nuclei, which structural morphology is unsuitable for further maturation of target anisotropic nanoparticle. This peculiarity of seed maturation was revealed previously in the kinetic studies conducted in [37,38]. It was shown that overexposure of nuclei leads to the formation of spherical and cubic particles. Such by-products are often present in the colloids of nanorods.

Distribution of the electromagnetic field on the surface of nanobones occurs differently in comparison with the nanorod surface. This reflects in the contour of the absorption spectrum of nanoparticles (Figure 2). For nanobones, the absorption spectrum is characterized by a new component at 570 nm located between the longitudinal and transverse modes of localized surface plasmon resonance. This feature appears due to the surface curvature in the “concave” regions. In these regions, the more significant localization and amplification of the electromagnetic field occur that allows achieving a higher enhancement of Raman scattering from the molecules located in such places.

Local amplification of the electromagnetic field on the nanobone edges occurs at any of the mutual orientation of the field and nanoparticle. When the nanobones are oriented along the electric field vector, localization of the electromagnetic field occurs on their edge petals similar to the nanorods. This leads to the appearance of the longitudinal plasmon resonance band in the absorption spectrum. At the orientation of nanobones across the electric field vector, localization of the electromagnetic field occurs on their long sides, providing the transverse plasmon resonance band in the absorption spectrum. Apart from that, the field is also concentrated in the regions of negative curvature on the short sides of the nanobones formed by two petals. In this dimple, the superposition of electromagnetic fields near two curvatures occurs, providing a stronger local field. This situation resembles the formation of hot spots in spherical nanoparticle dimers. Thus, considering the statistically equiprobable orientations of nanoparticles in a colloidal solution or in a biological sample, nanobones provide a higher SERS signal in comparison with nanorods, which has already been confirmed experimentally [39].

However, the distribution of the two lobes on the ends of the nanobones is similar to the dimeric nanostructure, which may bring about enhancement of the local electromagnetic field in the gap region between the lobes, causing SERS increased

**Figure 3:**
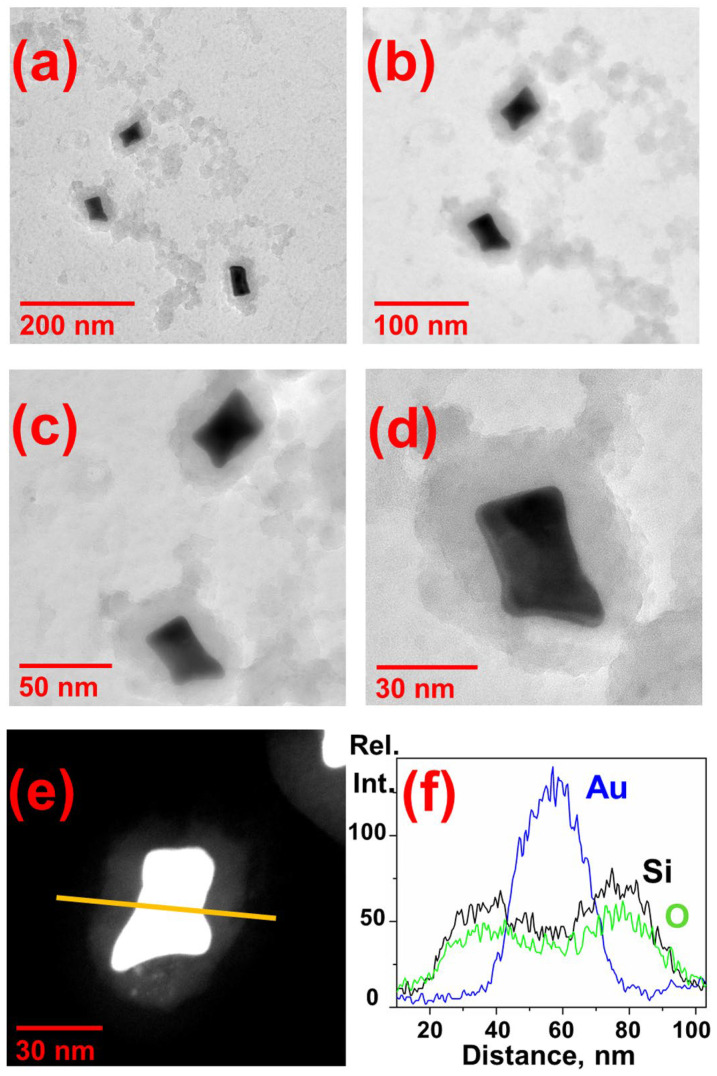
(**a**–**d**) TEM images of gold nanoparticles coated with silica using MPTMS. (**e**) STEM image of nanotag with an arrow indicating the EDX line scan position. (**f**) EDX line scan for Si Kα, O Kα, and Au Lα signals of the particle shown in (**e**).

Figure 3 shows the TEM and STEM images of gold nanobones coated with silica together with EDX line scan for Si Kα, O Kα, and Au Lα signals collected along the nanotag. EDX line scan clearly indicates silica surrounding a gold core. Apparently, the mechanism of dye-impregnated silica shell formation is as follows. At the first stage, MPTMS molecules bind to the surface of Au nanoparticles via thiol groups, displacing electrostatically immobilized CTAB molecules and making the surface vitreophilic. Further, added trimethoxysilane undergo hydrolysis under alkaline conditions with the formation of silanol groups transforming to -Si-O-Si- bonds. Trimethoxysilane fragments of previously attached MPTMS molecules serve as the template for the three dimensional polymerization of TEOS during its hydrolysis. It has been shown previously that silicon oxide shells formed on templated inorganic nanoparticles through alkaline hydrolysis have a mesoporous structure [40,41]. Thus, positively charged Cy5.5 amine added simultaneously with the onset of shell growth is able to encapsulate in the inner cavities of growing structure throughout the thickness. The thickness of the resulting shell is about 20 nm, providing the largest overall longitudinal particle size of about 65–70 nm, which fits the allowable sizes of nano-objects in biomedical applications suitable for intracellular endocytosis [42]. In particular, for silica particles with a diameter of 50–100 nm, an effective excretion from the intestine along the hepatobiliary pathway was shown to occur within 30 min after intravenous administration [43].

### 3.2. Study of Nanotag Intracellular Localization by Scanning Electron Microscopy

**Figure 4:**
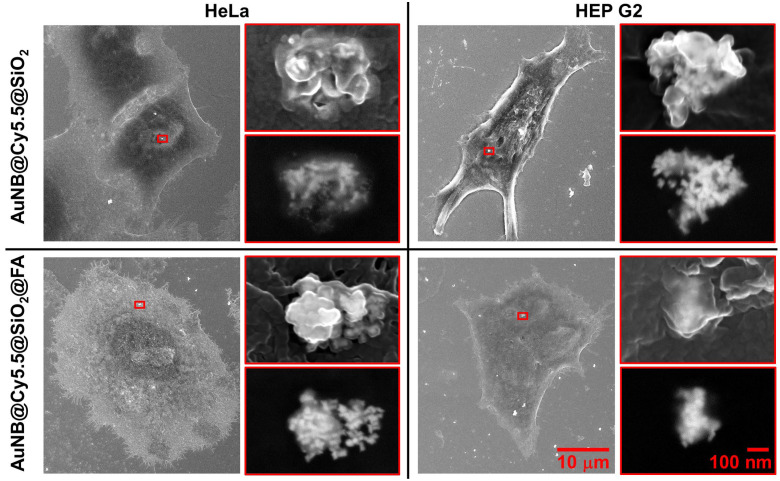
SEM images of HeLa and HEP G2 cells after incubation with nanotags. The large images show a general view of the cell in InLens mode. Two images on the right are the enlarged area with nanoparticle agglomerates highlighted with a red frame. The top image in each case was obtained in InLens mode, and the bottom image was obtained in AsB mode. All series have the same scale bar indicated bottom right.

The SEM images of the cell samples after incubation with the prepared nanotags are given in Figure 4. According to the obtained images, the nanoparticle behavior after interaction with cell membrane looks the same for epithelial (HeLa) and hepatocellular (HEP G2) carcinoma. No significant difference was also observed between the folate-modified and unmodified nanoparticles. Colloidal nanoparticles tend to be absorbed by cells in the form of agglomerates. Such behavior can be beneficial in terms of the “hot spots” effect in situ as well as to intensify the localized photothermal therapy [44,45]. Based on the comparison of images taken in AsB (shows heavy elements predominantly) and InLens (shows all elements) modes, it can be concluded that the cell membrane covers the nanoparticles which locate inside the cell at different depths. SEM does not provide information on the nanoparticle spatial distribution inside the cells and shows only agglomerates located not far from the surface. However, that is the only single method able to provide information about the form of the nanoparticles disposed under the cell membrane.

### 3.3. Nanotag SERS Response in the Solution Phase

**Figure 5:**
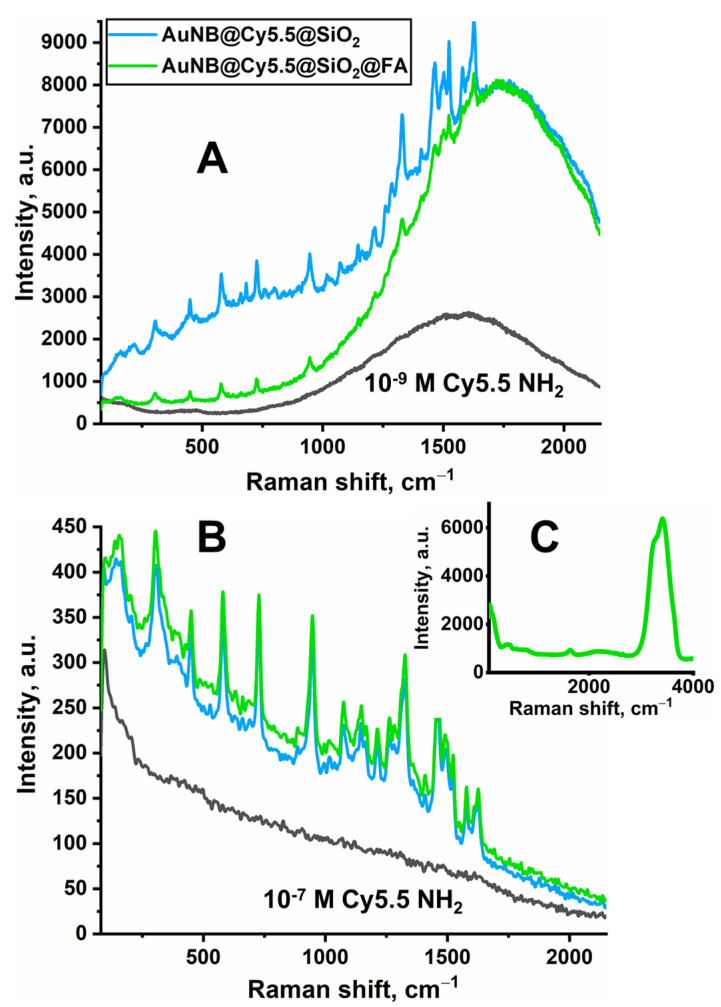
SERS spectra of nanotags colloidal solutions before and after functionalization with folic acid recorded under excitation with 632.8 nm (**A**), 785 nm, (**B**) and 532 nm (**C**) lasers. To show fluorescence nature of baseline in the SERS spectra, parts (**A**,**B**) include the spectra of cyanine 5.5 amine aqueous solutions obtained under the same acquisition parameters.

To predict the optical response of the obtained nanoparticles coated with silica, the SERS spectra of their colloidal solutions were recorded at excitation by several wavelengths (Figure 5). A pronounced Raman signal appears when using the lasers of 632.8 and 785 nm and is not observed when a green laser with a wavelength of 532 nm is used. This fact proves that the origin of the detected Raman signal is due to the nanoparticles’ plasmon resonance. It should be noted that Raman bands observed in the region of 1250–1750 cm^−1^ are more intense in comparison with the bands at lower wavenumbers in the spectrum excited by laser of 632.8 nm. This may evidence a resonance Raman scattering contribution since the absorption maximum of cyanine 5.5 amine locates at 684 nm. At higher wavelengths, when the SERS spectrum is excited by laser of 785 nm, the region of dye absorption does not overlap with the acquisition range, and a different distribution of relative intensities is observed (Figure 5B).

It should also be noted that the SERS signal from the nanotags modified by folic acid is significantly lower in comparison with the unmodified tags at excitation by 632.8 nm laser, wherein a comparable fluorescent background (a broad baseline band in the range of 1200–2000 cm^−1^) can be observed in both spectra. The mentioned difference in the intensities is not observed in the SERS spectra excited by 785 nm laser; moreover, a slight increase in intensity can be noted. It is possible to suggest that a partial agglomeration of particles takes place upon the modification by APTES and folic acid. The latter causes the red shift of the plasmon resonance band. Hence, this reduces Raman enhancement provided by the modified particles upon excitation of 632.8 nm and increases the SERS signal obtained upon excitation by laser of 785 nm. Such an effect has been well studied and even used to shift the plasmon resonance band to higher wavelengths and, thus, to increase the efficiency of photothermal therapy [46]. Finally, the observation of noticeable fluorescent background along with the SERS signal from the nanotags coated with silica indicates not only the presence of fluorophore near the surface but also its distribution throughout the shell. Taking into account that the shell thickness reaches 20 nm, it can be expected that a sufficient amount of the fluorophore could be incorporated into the pores of mesoporous silica at the distances where fluorescence quenching is minimized. The dependence of fluorescence quenching on the distance between the fluorophore molecule and plasmon core was studied earlier. In particular, it was shown for nanorods that fluorescence quenching is limited by a distance of approximately 2 nm from the core [4,28]. Performing three purifications at nanotag preparation (replacement of isopropanol with water, purification from excess APTES, and removing the excess unbound folic acid) guarantees that the observed fluorescence signal comes precisely from cyanine 5.5 amine incorporated into the silica shell but not from the fluorophore remaining in the solution.

### 3.4. Study of Nanotag Cell Imaging Performance by Surface-Enhanced Raman Spectroscopy

**Figure 6:**
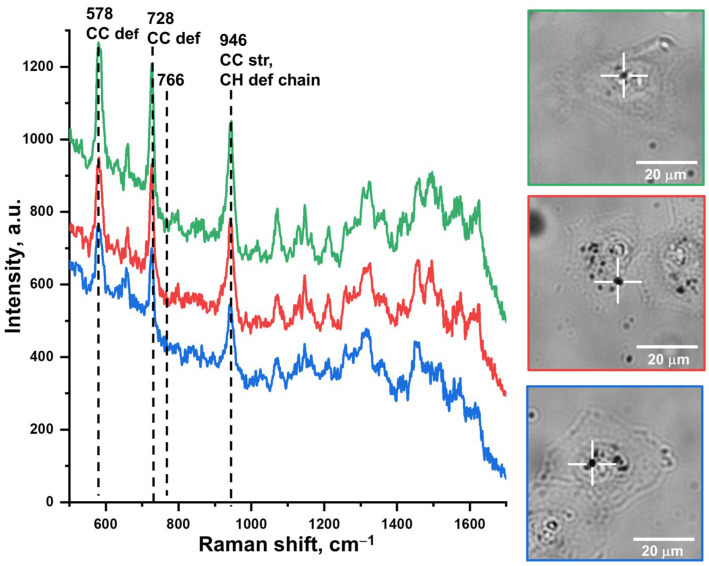
SERS spectra recorded from the nanoparticle agglomerates in different randomly selected HeLa cells. The inset shows the optical images of the studied cells and the points of spectra acquisition. In the bands description, def. means deformation and str. means stretching.

To select the acquisition conditions for cell imaging and assess the reproducibility, the SERS spectra were recorded from several points inside the cells related to nanoparticle agglomerates and visually observed in an optical microscope (Figure 6). These agglomerates were formed probably as the result of cellular endocytosis. Obviously, nanoparticles agglomeration provides the “hot spots” effect which enhances the Raman signal from the dye with a higher efficiency [47,48].

The contour of the recorded spectra fully corresponds to the spectra obtained for the nanotags in the colloid state. The intensities of vibrational bands showed a good reproducibility between different agglomerates located in various parts of a single cell. The conditions for recording the SERS spectra in the colloid state with excitation by laser of 785 nm (power of 25 mV, accumulation time of 4 s and two repetitions) turned out to be sufficient for constructing Raman images of cell monolayers after incubation with nanotags. These conditions ensured the minimum time spent on the acquisition of one point while maintaining a low laser power which does not cause the dye photodegradation.

**Figure 7:**
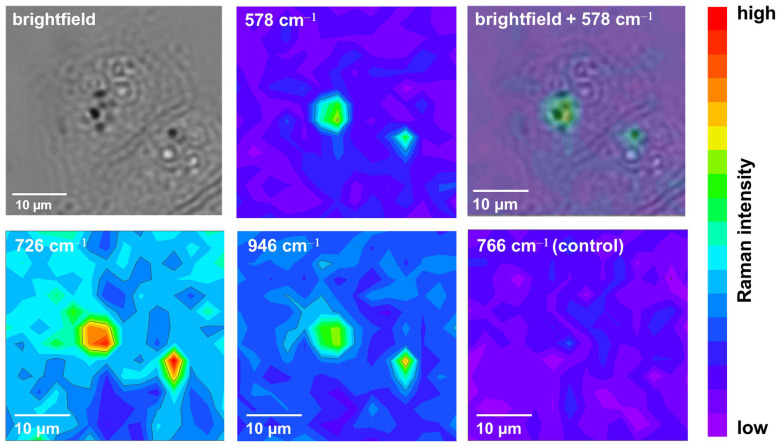
SERS images of two HeLa cells after incubation with nanotags.

SERS imaging of the cell samples was carried out in the mode of point-by-point spectra acquisition in the range of 457–1777 cm^−1^. The study of the cell samples was carried out on an area of 14*14 points with a step of 3 μm and took about one hour per image. SERS images were constructed using three different bands of cyanine 5.5 amine: 578, 726, and 946 cm^−1^ (Figure 7). The image based on a wavenumber of 766 cm^−1^ was also constructed as a control to demonstrate the absence of signal in accordance with the expectations. To eliminate a possible contribution of fluorescence from the dye, cellular components and quartz slide, the obtained spectra were processed using the asymmetric least squares smoothing function, which has become a reliable solution of the Raman spectra baseline problem [49]. The followed parameters of smoothing function were used: an asymmetry factor of 10^−4^, a threshold 10^−4^, a smoothing factor of 4, and 20 repeats. The images obtained with different spectral bands show a similar signal distribution, identifying the areas near the cell nuclei. Thus, the imaging function of the prepared nanotags is proved to be a reliable one.

### 3.5. Study of Nanotag Cytotoxity by Mitochondrial Reductase Activity Assay

**Figure 8:**
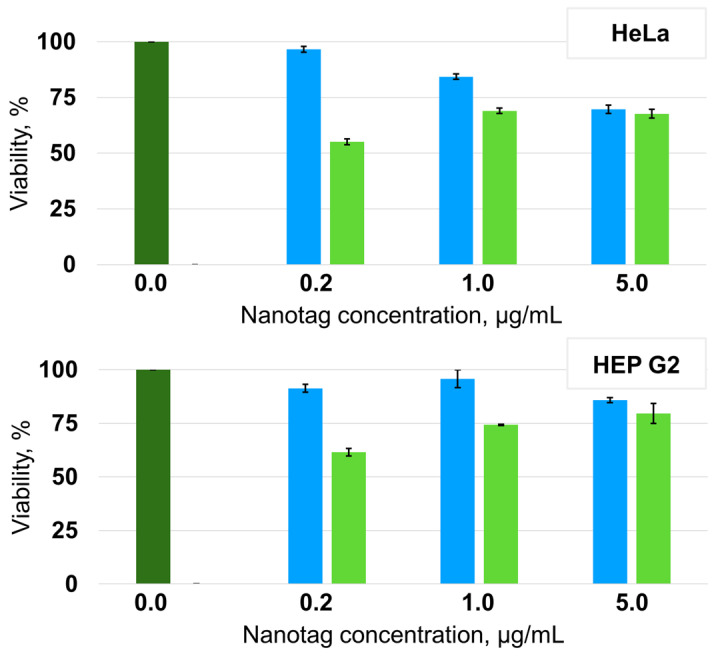
Viability of HeLa and HEP 2G cells after incubation with the nanotags before (blue) and after (green) functionalization with folic acid.

To evaluate the toxic and proliferative effect of the nanotags against human cells, an MTT test was performed with the folate-positive HeLa cell line and folate-negative HEP G2 cell line (Figure 8).

Both cell lines show the decrease in viability with the increase of the concentration of the nanotags non-functionalized with folic acid. This indicates clearly their dose-dependent toxicity profile. The nanotags overcome the threshold of cytotoxicity at a concentration higher than 1 μg/mL for HeLa cells. For HEP G2 line, it can be stated that there is no cytotoxicity of non-functionalized nanotags in the studied concentration range. The cell viability remains at a level higher than 80% compared with control cells without nanotags that is assumed to be acceptable [50]. The nanotags functionalized with folic acid demonstrate a completely different effect on the cells. For both lines, the increase of cell viability with the increase of nanotags concentration can be noticed, probably, revealing the activation of proliferative effect. Certainly, this finding requires further investigations.

In general, the HEP G2 line tolerates better the presence of nanoparticles of all types. This may be considered as a good sign because this line is a model line of hepatocytes participating in the hepatobiliary pathway. The increase in HEP G2 cell viability with the increase of the concentration of folate-functionalized nanotags can be explained by the induction of hepatocyte proliferation as a response on the presence of external stimulus. Similar behavior of hepatocytes was previously described for some nanoparticles with different surface structures [51].

## 4. Conclusions

In this work, we represented a novel procedure for the preparation of silica-coated gold nanostructures loaded with cyanine 5.5 for cell imaging with SERS spectroscopy. The described method using 4-mercaptopropyltrimethoxysilane covalent linker at silica coating is simple and significantly accelerated in comparison with known methods based on the recharge of gold surface with polyelectrolytes. The proposed procedure makes it possible to prepare the ready-to-use SERS nanotags in only two stages. The first stage includes the optimized protocol of functionalization the CTAB-stabilized gold nanoparticles of bone-like shape with MPTMS, the adjusted concentration of which in the reaction mixture does not induce the nanoparticle agglomeration observed previously. The second stage includes the silica shell growth, fluorophore impregnation, and shell surface pre-functionalization for delivery vector which are performed in a one-pot regime, i.e., without intermediate stages of isolation and purification.

Via TEM and EDX analysis, it was proved that the obtained nanotags have the anisotropic core-shell structure, average size of 50×70 nm, and average silica shell thickness of about 20 nm, meeting the criteria of cell endocytosis. It is demonstrated that encapsulated in silica fluorophore distributes throughout the shell due to the applied synthetic route and is capable of emitting both SERS and fluorescence signals. The SERS studies with cell samples showed the high signal level and good reproducibility from the nanotags uptaken by the cells under fairly mild exposure conditions (excitation wavelength of 785 nm, power of 25 mV, 8 s per point). The obtained nanotags reduce the viability of folate-positive HeLa cancer cells more significantly in comparison with folate-negative HEP G2 cells even under dark-mode cytotoxicity test conditions. In general, the obtained nanotags are promising candidates for further research addressed to localized photothermal therapy for cancer.

## Data Availability

The data presented in this study are available on request from the corresponding author.

## References

[1] Tahir M.A., Dina N.E., Cheng H., Valev V.K., Zhang L. (2021). Surface-enhanced Raman spectroscopy for bioanalysis and diagnosis. Nanoscale.

[2] Meir R., Motiei M., Popovtzer R. (2014). Gold nanoparticles for in vivo cell tracking. Nanomedicine.

[3] Xu Y., Wang H., Zhang M., Zhang J., Yan W. (2021). Plasmon-enhanced antibacterial activity of chiral gold nanoparticles and in vivo therapeutic effect. Nanomaterials.

[4] Bian Y., Liu S., Zhang Y., Liu Y., Yang X., Lou S., Wu E., Wu B., Zhang X., Jin Q. (2021). Distance-Dependent Plasmon-Enhanced Fluorescence of Submonolayer Rhodamine 6G by Gold Nanoparticles. Nanoscale Res. Lett..

[5] Ahamad N., Al-Amin M.D., Ianoul A. (2013). Distance dependent surface enhanced Raman and fluorescence by supported 2D assembly of plasmonic metal nanoparticles. Asian J. Chem..

[6] Kennedy B.J., Spaeth S., Dickey M., Carron K.T. (1999). Determination of the Distance Dependence and Experimental Effects for Modified SERS Substrates Based on Self-Assembled Monolayers Formed Using Alkanethiols. J. Phys. Chem. B.

[7] Le Ru E.C., Blackie E., Meyer M., Etchegoint P.G. (2007). Surface Enhanced Raman Scattering Enhancement Factors:  A Comprehensive Study. J. Phys. Chem. C.

[8] Maher R.C., Cohen L.F., Le Ru E.C., Etchegoin P.G. (2006). On the experimental estimation of Surface Enhanced Raman Scattering (SERS) cross sections by vibrational pumping. J. Phys. Chem. B.

[9] Lee J.M., Kim S.O. (2016). Enhancing Organic Solar Cells with Plasmonic Materials. ChemNanoMat..

[10] Vines J.B., Yoon J.-H., Ryu N.-E., Lim D.-J., Park H. (2019). Gold Nanoparticles for Photothermal Cancer Therapy. Front. Chem..

[11] Gharatepe A., Salehi R. (2017). Recent progress in theranostic applications of hybrid gold nanoparticles. Eur. J. Med. Chem..

[12] Sanchis-Gual R., Coronado-Puchau M., Mallah T., Coronado E. (2023). Hybrid nanostructures based on gold nanoparticles and functional coordination polymers: Chemistry, physics and applications in biomedicine, catalysis and magnetism. Coord. Chem. Rev..

[13] Maccora D., Dini V., Battocchio C., Fratoddi I., Cartoni A., Rotili D., Castagnola M., Faccini R., Bruno I., Scotognella T. (2019). Gold nanoparticles and nanorods in nuclear medicine: A mini review. Appl. Sci..

[14] Mohajer F., Ziarani G.M., Badiei A. (2021). New advances on Au–magnetic organic hybrid core–shells in MRI, CT imaging, and drug deliver. RSC Adv..

[15] Arsalani S., Arsalani S., Isikawa M., Guidelli E.J., Mazon E.E., Ramos A.P., Bakuzis A., Pavan T.Z., Baffa O., Carneiro A.A.O. (2023). Hybrid Nanoparticles of Citrate-Coated Manganese Ferrite and Gold Nanorods in Magneto-Optical Imaging and Thermal Therapy. Nanomaterials.

[16] Elmi G.R., Saleem K., Baig M.M.F.A., Aamir M.N., Wang M., Gao X., Abbas M., Rehman M.U. (2022). Recent Advances of Magnetic Gold Hybrids and Nanocomposites, and Their Potential Biological Applications. Magnetochemistry.

[17] Li X., Zhang Y., Liu G.K., Luo Z., Zhou L., Xue Y., Liu M. (2022). Recent progress in the applications of gold-based nanoparticles towards tumor-targeted imaging and therapy. RSC Adv..

[18] Fratoddi I., Venditti I., Battocchio C., Carlini L., Amatori S., Porchia M., Tisato F., Bondino F., Magnano E., Pellei M. (2019). Highly hydrophilic gold nanoparticles as carrier for anticancer copper(I) complexes: Loading and release studies for biomedical applications. Nanomaterials.

[19] Bavelaar B.M., Song L., Jackson M.R., Able S., Tietz O., Skaripa-Koukelli I., Waghorn P.A., Gill M.R., Carlisle R.C., Tarsounas M. (2021). Oligonucleotide-Functionalized Gold Nanoparticles for Synchronous Telomerase Inhibition, Radiosensitization, and Delivery of Theranostic Radionuclides. Mol. Pharm..

[20] Kumar S., Mongia A., Gulati S., Singh P., Diwan A., Shukla S. (2020). Emerging theranostic gold nanostructures to combat cancer: Novel probes for Combinatorial Immunotherapy and Photothermal Therapy. Cancer. Treat. Res. Commun..

[21] Moreira A.F., Rodrigues C.F., Reis C.A., Costa E.C., Correia I.J. (2018). Gold-core silica shell nanoparticles application in imaging and therapy: A review. Micropor. Mesopor. Mater..

[22] Rascol E., Daurat M., Da Silva A., Maynadier M., Dorandeu C., Charnay C., Garcia M., Lai-Kee-Him J., Bron P., Auffan M. (2017). Biological fate of Fe_3_O_4_ core-shell mesoporous silica nanoparticles depending on particle surface chemistry. Nanomaterials.

[23] Ko J.A., Lim H.B. (2013). Metal/dye-doped core-shell silica nanoparticles for potential use in bioassay. J. Anal. At. Spectrom..

[24] Wang P., Qu Y., Li C., Yin L., Shen C., Chen W., Yang S., Bian X., Fang D. (2015). Bio-functionalized dense-silica nanoparticles for MR/NIRF imaging of CD146 in gastric cancer. Int. J. Nanomed..

[25] Badruddoza A.Z.M., Rahman T., Ghosh S., Hossain Z., Shi J., Hidajat K., Uddin M.S. (2013). β-Cyclodextrin conjugated magnetic, fluorescent silica core-shell nanoparticles for biomedical applications. Carbohydr. Polym..

[26] Huang P., Bao L., Zhang C., Lin J., Luo T., Yang D., He M., Li Z., Gao G., Gao B. (2011). Folic acid-conjugated silica-modified gold nanorods for X-ray/CT imaging-guided dual-mode radiation and photo-thermal therapy. Biomaterials..

[27] Nguyen N.H., Tran D.L., Truong-Thi N., Nguyen C.K., Tran C.T., Nguyen D.H. (2022). Simply and effectively control the shell thickness of hollow mesoporous silica nanoparticles by polyethylene glycol for drug delivery applications. J. Appl. Polym. Sci..

[28] Chen B., Chen C., Tao C., Zeng H., Zhang L., Yang M., Han Z. (2017). Size and distance dependent fluorescence enhancement of nanoporous gold. Opt. Express.

[29] Graf C., Vossen D.L.J., Imhof A., van Blaaderen A.A. (2003). General Method to Coat Colloidal Particles with Silica. Langmuir.

[30] Stober W., Fink A., Bohn E. (1968). Controlled growth of monodisperse silica spheres in the micron size range. J. Colloid Interface Sci..

[31] Rosemary M.J., Maclaren I., Pradeep T. (2004). Carbon onions within silica nanoshells. Carbon.

[32] Lee S., Kwon J.A., Park K.H., Jin C.M., Joo J.B., Choi I. (2018). Controlled drug release with surface-capped mesoporous silica nanoparticles and its label-free in situ Raman monitoring. Eur. J. Pharm. Biopharm..

[33] Pastoriza-Santos I., Perez-Juste J., Liz-Marzan L.M. (2006). Silica-coating and hydrophobation of ctab-stabilized gold nanorods. Chem. Mater..

[34] Perez-Juste J., Pastoriza-Santos I., Liz-Marzan L.M. (2005). Gold nanorods: Synthesis, characterization and applications. Coord. Chem. Rev..

[35] Scarabelli L., Sánchez-Iglesias A., Pérez-Juste J., Liz-Marzán L.M. (2015). A “Tips and Tricks” Practical Guide to the Synthesis of Gold Nanorods. J. Phys. Chem. Lett..

[36] Yoshida A., Uchida N., Kometani N. (2009). Synthesis and Spectroscopic Studies of Composite Gold Nanorods with a Double-Shell Structure Composed of Spacer and Cyanine Dye J-Aggregate Layers. Langmuir.

[37] Park K., Drummy L.F., Wadams R.C., Koerner H., Nepal D., Fabris L., Vaia R.A. (2013). Growth mechanism of gold nanorods. Chem. Mater..

[38] Watt J., Hance B.G., Anderson R.S., Huber D.L. (2015). Effect of Seed Age on Gold Nanorod Formation: A Microfluidic, Real-Time Investigation. Chem. Mater..

[39] Zhu J., Zhang Q., Zhang C.-H., Weng G.-J., Zhao J., Li J.-J., Zhao J.-W. (2017). Synthesis of colloidal gold nanobones with tunable negative curvatures at end surface and their application in SERS. J. Nanoparticle Res..

[40] Gorelikov I., Matsuura N. (2008). Single-Step Coating of Mesoporous Silica on Cetyltrimethyl Ammonium Bromide-Capped Nanoparticles. Nano Lett..

[41] Yoon S.B., Kim J.-Y., Kim J.H., Park Y.J., Yoon K.R., Park S.-K., Yu J.-S. (2007). Synthesis of monodisperse spherical silica particles with solid core and mesoporous shell: Mesopore channels perpendicular to the surface. J. Mat. Chem..

[42] Hirn S., Semmler-Behnke M., Schleh C., Wenk A., Lipka J., Schäffler M., Takenaka S., Möller W., Schmid G., Simon U. (2011). Particle size-dependent and surface charge-dependent biodistribution of gold nanoparticles after intravenous administration. Eur. J. Pharm. Biopharm..

[43] Takeuchi I., Nobata S., Oiri N., Tomoda K., Makino K. (2017). Biodistribution and excretion of colloidal gold nanoparticles after intravenous injection: Effects of particle size. Biomed. Mater. Eng..

[44] Solovyeva E.V., Smirnov A.N., Svinko V.O., Strelnikov A.S., Shevchuk A.I., Kazarian S.G. (2022). Unraveling a role of molecular linker in nanoparticles self-organization by SERS spectroscopy: Comparative study of three aromatic diamines. Colloids Surf. A Physicochem. Eng. Asp..

[45] Park S., Kim H., Lim S.C., Lim K., Lee E.S., Oh K.T., Choi H.G., Youn Y.S. (2019). Gold nanocluster-loaded hybrid albumin nanoparticles with fluorescence-based optical visualization and photothermal conversion for tumor detection/ablation. J. Control. Release.

[46] Marghani B.H., Fehaid A., Ateya A.I., Ezz M.A., Saleh R.M. (2022). Photothermal therapeutic potency of plasmonic silver nanoparticles for apoptosis and anti-angiogenesis in testosterone induced benign prostate hyperplasia in rats. Life Sci..

[47] Shiohara A., Wang Y., Liz-Marzán L.M. (2014). Recent approaches toward creation of hot spots for SERS detection. J. Photochem. Photobiol. C Photochem. Rev..

[48] Svinko V.O., Shevchuk A.I., Smirnov A.N., Makeeva D.V., Solovyeva E.V. (2022). Gold nanostars-based labels for surface-enhanced Raman scattering imaging with red medical lasers. Opt. Spectrosc..

[49] He S., Zhang W., Liu L., Huang Y., He J., Xie W., Wu P., Du C. (2014). Baseline correction for Raman spectra using an improved asymmetric least squares method. Anal. Methods.

[50] Akal Z.U., Alpsoy L., Baykal A. (2016). Superparamagnetic Iron Oxide Conjugated with Folic acid and Carboxylated Quercetin for Chemotherapy applications. Ceram. Int..

[51] Sun Q., Kanehira K., Taniguchi A. (2016). Low doses of TiO_2_-polyethylene glycol nanoparticles stimulate proliferation of hepatocyte cells. Sci. Technol. Adv. Mater..

